# Supplementary material for: Magnitude of malnutrition and its associated factors among pediatric cancer patients on chemotherapy at oncology centers in Addis Ababa, Ethiopia, 2024
Source: PLoS One. 2026 Feb 18;21(2):e0342544. doi: 10.1371/journal.pone.0342544 (PMC12915981; doi:10.1371/journal.pone.0342544)
Supplement: Attachment — Submitted filename: Response to Reviewer.docx [file pone.0342544.s002.docx]

**Response to Reviewers**

**To:** Syeda Humaida Hasan, Diploma in Child Health, FCPS (Pediatrics), Academic Editor, PLOS ONE, and the Esteemed Reviewers

**From:** Habtamu Wondmagegn (Corresponding Author)

**Re:** Revision of Manuscript PONE-D-25-44535 – "**Magnitude of Malnutrition and Its Associated Factors among Pediatric Cancer Patients on Chemotherapy at Oncology Centers in Addis Ababa, Ethiopia, 2024**."

Dear Dr. Hasan and Reviewers,

We would like sincerely thank you for the constructive feedback and the opportunity to revise our manuscript. The comments we have received have been helpful to improve the quality, clarity, and scientific rigor of our work. We have carefully addressed each point raised, and the changes are detailed in a point-by-point manner below.

All revisions in the manuscript are highlighted in the 'Revised Manuscript with Track Changes' file. We believe the manuscript has been greatly improved and now fully meets the publication standards of PLOS ONE.

**Response to Journal Requirements**

1. **Style Requirements:** We have reformatted the entire manuscript to comply with the PLOS ONE style templates.
2. **Data Availability:** The dataset includes sensitive participant information and cannot be publicly shared due to ethical restrictions approved by the Institutional Review Board (IRB) of Arba Minch University, as well as the terms of informed consent signed by study participants. Public disclosure would compromise participant privacy and confidentiality. However, de-identified data may be made available to qualified researchers upon reasonable request. Such requests will be subject to review and approval by the IRB of Arba Minch University. Data access requests may be directed to the Institutional Review Board (IRB) of the College of Medicine and Health Sciences, Arba Minch University (Email: [irb@amu.edu.et](mailto:irb@amu.edu.et)).
3. **Figure Caption:** A descriptive caption has been added for Figure 2.
4. **Citation of Recommended Works:** We have reviewed the literature suggested by the reviewers and have incorporated relevant citations to improve the context and depth of our discussion.

**Response to Additional Editor Comments**

**Comment 1: Sample size calculation: Clarify why the “proportion of pregnancy within 12 months” was used in the sample size estimation. Explain its relevance to the study outcome and provide a supporting reference.**

**Response:** We apologize for this confusing error. The mention of "proportion of pregnancy" was a mistake during the write-up and is entirely irrelevant to our study. We have removed it in the revised version. The sample size was correctly calculated based on the proportion of malnutrition (p=0.449) from the referenced Jimma study (Ref 11). We have made revisions the 'Sample size determination' section for accuracy and clarity, as detailed in our response to Reviewer #1.

**Comment 2: Duration of cancer: The inclusion criterion of “duration of cancer less than 2 years” appears to include newly diagnosed cases (<1 month). In such cases, malnutrition may result from the malignancy itself rather than its treatment or chronic course. Please revise or justify this criterion in line with your study objectives.**

**Response:** This is a very insightful point. We have clarified in the 'Methods' section (lines 178-179) that "duration of cancer" refers to the "time since diagnosis." We agree that in cross-sectional studies like ours, it is challenging to determine whether malnutrition is a consequence of the pre-disease status, the cancer itself, or the treatment. To specifically address this, we added a sentence to the 'Limitations' section (lines 383-385): *"Furthermore, the cross-sectional nature of this study limits the ability to establish causality and to distinguish whether the observed malnutrition was pre-existing, a consequence of the cancer itself, or a result of chemotherapy treatment."*

**Comment 3: Tables and demographic variables: Tables should be formatted more clearly. For example, “marital status” should be specified as “marital status of the mother.” whereas the inclusion of “birth interval” is not clearly useful. If you choose to retain it, please clarify its relevance to the study.**

**Response:** We appreciate the editor's recommendation.

- We have revised Table 1 for clarity. "Marital status" is now specified as "Mother's marital status."
- Since "birth interval" is a known demographic feature linked to child nutrition in our scenario, we have kept it. We have added a brief justification for its inclusion in the 'Methods' section under 'Data Collection' (lines 155-156): *"Socio-demographic variables such as maternal education, household income, and birth interval were collected as they are established distal determinants of child nutritional status in the literature."*

**Response to Reviewers' Comments**

**Reviewer 1**

We sincerely thank Reviewer #1 for their valuable and technical feedback, which has greatly improved the methodological transparency of our manuscript.

**Comment 1 (Line 123): The equation is difficult to understand. Please explain the variables with more details. Apparently the P and p represent the same variable, and the meaning of it on line 125 does not make sense to me. Furthermore, the result of the equation does not equal to the result of the Authors.**

**Response:** We sincerely apologize for these errors and the lack of clarity. We have completely rewritten this section.

- We have standardized the variable for proportion to p and clearly defined all variables (n, Zα/2, p, d) below the formula.
- We have corrected the calculation. The initial calculation of 301 was incorrect. The revised calculation is (1.96) ² * (0.449 * 0.551) / (0.055) ² ≈ 314. With a 10% non-response rate, the target was **345**. We enrolled **320** participants, which provides a margin of error of ±5.6%, which is still precise and acceptable. Please see the revised 'Sample size determination' section (lines 120-133).

**Comment 2 (Line 364): Many of these Abbreviations are not referenced in the main text. In addition, some concepts need to be explained, e.g., AOR and COR.**

**Response:** Thank you for pointing this out.

- After going over the list of abbreviations, we eliminated those that weren't used in the paper (such as AML, BL, HL, HFA, and WFH). Please see this at (line 396 – 403).
- We have defined **AOR (Adjusted Odds Ratio)** and **COR (Crude Odds Ratio)** upon their first use in the 'Data analysis' section (lines 190-191): *"To assess the strength of association, Crude Odds Ratios (COR) and Adjusted Odds Ratios (AOR) with 95% CIs were computed."*

**Comment 3: In general, the Authors should provide more technical details on how the models were fitted.**

**Response:** We have expanded the 'Data analysis' section (lines 182-194) to provide more detail:
"*Both bivariable and multivariable binary logistic regression models were fitted. The model-building process involved entering all independent variables into the bivariable analysis. Variables with a p-value < 0.25 in the bivariable analysis were selected as candidates for the multivariable model. The multivariable model was then constructed using a backward stepwise likelihood ratio method to identify factors independently associated with malnutrition. The goodness-of-fit of the final model was assessed using the Hosmer-Lemeshow test, and the presence of multicollinearity among the independent variables was evaluated using the Variance Inflation Factor (VIF), with a value of 10 used as a threshold for serious multicollinearity."*

**Reviewer 2**

We appreciate Dr Valérie Marcil's insightful and helpful feedback, which greatly improved our manuscript's conceptual framework and clarity.

**Comment 1: As of today, there is still no consensus on the best definition of pediatric malnutrition... Why do they use only these parameters?... Why don't they assess stunting (height-for-age) as well?... Parameters should be identified in the abstract... argue their choice of tools...**

**Response:** This is a critical point, and we have substantially revised the manuscript to address it. We now revised and added a clarification in the methods, result, abstract and result section on the revised manuscript.

- **Methods:** We have added a justification in the 'Measurements' section (lines 158-165): *"Nutritional status was assessed using WHO Anthro software. For this study, undernutrition (wasting and underweight) was defined using Weight-for-Length/Height and BMI-for-age z-scores, as these are sensitive indicators of acute nutritional status, which is a primary concern in the context of active chemotherapy. Stunting (Height-for-Age z-score < -2 SD) was also assessed as an indicator of chronic malnutrition, and its prevalence is reported separately in the results."*
- **Results:** We have added the prevalence of stunting to the 'Magnitude of malnutrition' section (line 219-221): *"The prevalence of wasting/low BMI-for-age was 28.5%. Additionally, the prevalence of stunting,* *based on height-for-age, was 30.5%."*
- **Abstract:** We have specified the parameters used (line 34-36): *"...the magnitude of malnutrition, defined by low BMI-for-age or weight-for-height/length z-scores..."*
- **Discussion:** We have added a new paragraph (lines 365-371) discussing our choice of tools and their limitations: *"The choice of anthropometric tools influences the reported prevalence of malnutrition. Our study primarily used BMI-for-age and weight-for-height to define malnutrition, focusing on acute weight loss and wasting, which are critical in acutely ill patients. This may underrepresent children with sarcopenia or fluid retention. The high prevalence of stunting we observed suggests a significant burden of chronic malnutrition, which may predate the cancer diagnosis. Future studies in this population would benefit from incorporating composite tools like MUAC and TSFT, or functional assessments, to provide a more comprehensive nutritional picture."*

**Comment 2: Authors present that presence of comorbidities was associated with malnutrition. These comorbidities are not defined in the methods sections. What are those comorbidities? How were they identified/defined?**

**Response:** Thank you. In the revised manuscript we defined "comorbidities" in the 'Methods' section under 'Data Collection' (lines 174–177): "*Comorbidities were defined as any other chronic health condition that coexisted with the cancer diagnosis. A study of the patient's medical chart revealed illnesses such as tuberculosis, HIV, congenital heart disease, and chronic kidney failure”*.

**Comment 3: Writing and English must be revised. They are too many sentences to state here. For example: ‘’Children with 2-4 years’ duration of cancer were significantly associated with being malnourished.’’**

**Response:** We thoroughly review the entire article to remove grammatical faults and increase clarity. The sentence highlighted has been corrected to: *"A cancer duration of 2-4 years was significantly associated with malnutrition.*" (Line 261, 269,388.,). We have also engaged a colleague proficient in academic English to conduct a final evaluation.

**Comment 4: ''Children form Households which earn <2000 a month'': precise the currency**

**Response:** We have added "ETB" (Ethiopian Birr) throughout the manuscript where income is mentioned (e.g., Abstract, Results, Tables 1 & 3).

**Comment 5: Methods: the yearly pool of pediatric cancer patients should be given for each center.**

**Response:** We have added an estimate to the 'Study Area' section (lines 108-110): "*Based on the daily and weekly attendance figures, it is estimated that the two centers collectively serve a pool of approximately 1,200-1,500 pediatric oncology patients annually, making them the primary referral centers for pediatric cancer in Ethiopia."*

**Conclusion**

We really appreciate the editor and reviewers' effort and expertise. We feel that by addressing all of the issues mentioned, we have considerably improved the quality and impact of our paper. We hope that the amended version is now considered appropriate for publishing in PLOS ONE.

Sincerely,

Habtamu Wondmagegn, MSc
On behalf of all co-authors.
