# Supplementary material for: Magnitude of malnutrition and its associated factors among pediatric cancer patients on chemotherapy at oncology centers in Addis Ababa, Ethiopia, 2024
Source: PLoS One. 2026 Feb 18;21(2):e0342544. doi: 10.1371/journal.pone.0342544 (PMC12915981; doi:10.1371/journal.pone.0342544)
Supplement: Attachment — Submitted filename: Response to Reviewers.docx [file pone.0342544.s003.docx]

**Response to Reviewers**

**To:** Syeda Humaida Hasan, Diploma in Child Health, FCPS (Pediatrics), Academic Editor, PLOS ONE

**From:** Habtamu Wondmagegn (Corresponding Author)

**Re:** Revision of Manuscript PONE-D-25-44535 – "**Magnitude of Malnutrition and Its Associated Factors among Pediatric Cancer Patients on Chemotherapy at Oncology Centers in Addis Ababa, Ethiopia, 2024**."

Dear Dr. Hasan,

We would like sincerely thank you for the constructive feedback and the opportunity to revise our manuscript. The comments we have received have been helpful to improve the quality, clarity, and scientific rigor of our work. We have carefully addressed each point raised, and the changes are detailed in a point-by-point manner below.

All revisions in the manuscript are highlighted in the 'Revised Manuscript with Track Changes' file. We believe the manuscript has been greatly improved and now fully meets the publication standards of PLOS ONE.

**Editor’s Point 1. Please re-write the operational definition of normal nutrition and malnutrition with clarity.**

**Response:** Thank you, the definitions have been clarified in the Operational Definitions section to specify that status is determined based on age-appropriate indicators. Please find this on line number 164-168.

**Editor’s Point 2. If a 95% confidence interval is used for weight-for-height, the same should also be reported for height-for-age. Please ensure consistency by correcting this in the Results section, Abstract, and Discussion.**

**Response:** Thank you, the 95% confidence interval for stunting (height-for-age) has been calculated and added to the Abstract, Results, and Discussion sections. Please find this on line number 35, 217,236, and 283

**Editor’s Point 3. It is preferable to report absolute numbers alongside percentages throughout the manuscript. Please revise accordingly.**

**Response:** Thank you, absolute numbers (n/N) have been added alongside percentages in the text. For example: The magnitude of malnutrition was 28.4% (n=91/320). The prevalence of stunting was 30.6% (n=98/320). Please find this on line number 235, 236, 282, and 283.

**Editor’s Point 4. Please rewrite lines 239–240 and 267–279. The language is repetitive and monotonous, and there is scope for improvement in writing style, variation in sentence structure, and clarity. Additionally, please review punctuation and capitalization carefully, particularly around line 339.**

**Response:** Thank you, we have thoroughly revised the indicated sections to improve the writing style, vary sentence structure, and enhance clarity. We have eliminated monotonous phrasing and ensured a more professional, academic tone. Additionally, we have carefully reviewed the entire manuscript for punctuation and capitalization errors. Please find this on line number 235- 238, 260 – 277.

**Editor’s Point 5. The Conclusion section should be rewritten in clear, standard English to better reflect the study findings.**

**Response:** Thank you for your comment. The conclusion has been refined for clarity and impact: as follows;” *In conclusion, this study demonstrates a high prevalence of malnutrition (28.4%) among pediatric cancer patients undergoing chemotherapy in Addis Ababa. Adolescents (11–15 years), children from households facing extreme poverty, those with hematologic malignancies, and those with swallowing difficulties were identified as being at the highest risk. These findings highlight the urgent necessity for routine nutritional screening and integrated support interventions in pediatric oncology centers to improve treatment efficacy and survival.”*

**In the tables:

Editor’s Point 6. Please change “age of a children” to “age of children.”**

**Response:** Thank you, we have updated from "Age of a children" to "Age of children".

**Editor’s Point 7. Correct the use of the apostrophe in “mothers’ marital status.”**

**Response:** Thank you, we have updated from“mothers’ marital status’’ to “mother’s marital status.”

**Editor’s Point 8. In the logistic regression analysis, please explain why “no swallowing difficulty” was not assessed against “swallowing difficulty” as the reference category. This requires clarification in the Methods or Results section.**

**Response:** We are very grateful to the Editor for identifying this discrepancy. Upon thorough review, we found that the rows for this variable in Table 3 were completely mislabeled in the previous version. The labels "Yes" and "No" (now updated to "Present" and "Absent") were inadvertently swapped, and the reference category was incorrectly assigned in the table layout.

We have corrected Table 3 in the revised manuscript. We now use **"Absent"** (no swallowing difficulty) as the reference category (AOR = 1.0). Consequently, the data now correctly shows that children with the **"Present"** status for swallowing difficulty have a significantly higher risk of malnutrition (AOR = 2.11, 95\% CI: 1.22–3.95), which is consistent with our results text, discussion, and clinical logic.

**Editor’s Point 9. In the Discussion section, please ensure that all key findings are adequately explained and compared with results from similar studies. This section still requires further improvement in depth and interpretation.**

Response: Thank you, we have extensively revised the Discussion section to provide greater scientific depth and clinical interpretation. Rather than simply listing comparative prevalence rates, we have now contextualized our findings within the unique socioeconomic and clinical landscape of Ethiopia. Please find this in line number 280-348

**Editor’s Point 10. Please note that data availability is a prerequisite for PLOS publications. As previously requested, the authors must share an anonymized dataset after obtaining permission from the appropriate authority and include a proper Data Availability statement.**

**Response:** We have updated our Data Availability statement to comply with PLOS ONE requirements. Ethical clearance for this study was obtained from the Institutional Review Board (IRB) of Saint Paul’s Millennium Medical College (SPHMMC). Because the dataset contains sensitive clinical information regarding pediatric oncology patients, the data are available upon reasonable request from the SPHMMC IRB for researchers who meet the criteria for access to confidential data. Please find this in line number 386- 390.

Interested researchers may contact the IRB via email at: **irb@sphmmc.edu.et**.
